# The effect of soil-borne pathogens depends on the abundance of host tree species

**DOI:** 10.1038/ncomms10017

**Published:** 2015-12-03

**Authors:** Yu Liu, Suqin Fang, Peter Chesson, Fangliang He

**Affiliations:** 1 SYSU-Alberta Joint Lab for Biodiversity Conservation, Sun Yat-sen University, Guangzhou 510275, China; 2 Guangdong Key Laboratory for Biodiversity Dynamics and Conservation, and State Key Laboratory of Biocontrol, School of Life Sciences, Sun Yat-sen University, Guangzhou 510275, China; 3 Department of Ecology and Evolutionary Biology, The University of Arizona, Tucson, Arizona 85721, USA; 4 Department of Life Sciences, National Chung Hsing University, Taichung 402, Taiwan; 5 Department of Renewable Resources, University of Alberta, 114St-89 Avenue, Edmonton, Alberta, Canada T6G 2H1

## Abstract

The overarching issue for understanding biodiversity maintenance is how fitness advantages accrue to a species as it becomes rare, as this is the defining feature of stable coexistence mechanisms. Without these fitness advantages, average fitness differences between species will lead to exclusion. However, empirical evidence is lacking, especially for forests, due to the difficulty of manipulating density on a large-enough scale. Here we took advantage of naturally occurring contrasts in abundance between sites of a subtropical tree species, *Ormosia glaberrima*, to demonstrate how low-density fitness advantages accrue by the Janzen–Connell mechanism. The results showed that soil pathogens suppressed seedling recruitment of *O. glaberrima* when it is abundant but had little effect on the seedlings when it is at low density due to the lack of pathogens. The difference in seedling survival between abundant and low-density sites demonstrates strong dependence of pathogenic effect on the abundance of host species.

## Abstract (web summary)

Fitness advantages conferred on species living at low density is thought to be one mechanism by which stable biodiversity is maintained. Here, Liu *et al*. show that recruitment of seedlings in high-density populations of a subtropical tree is suppressed by soil pathogens, with little effect at low-density.

## Graphical abstract

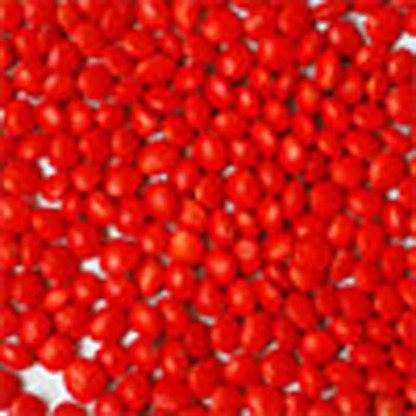

Stabilization mechanisms are essential for maintaining diversity in natural communities. Such mechanisms require the per capita growth rate of a population to be a decreasing function of abundance so that a species is able to increase at low abundance. This outcome forms the basis of the invasibility criterion^1^,^2^,^3^ that is critical for stable species coexistence regardless of the specific mechanisms applying to a community^2^,^4^,^5^. However, in most communities, there is a lack of understanding of the actual mechanisms contributing to this outcome, which is rarely rigorously demonstrated in nature^2^,^6^.

The decreasing function of per capita population growth with abundance that reflects fitness advantages at low density inversely implies also disadvantages at high density. This high-density disadvantage prevents individual species from overwhelming other species and promotes species diversity in communities. These advantages at low density and disadvantages at high density appear from the perspective of an individual species as negative density dependence (NDD) but reflect stabilizing niche differences between species in a community^1^, and in some older literature have been referred to as a community compensatory trend (CCT)^7^,^8^. However, controversy arises from recent studies about the strength of NDD in rare species versus common species, which appears to contradict the predictions of the CCT^9^,^10^,^11^. Although this controversy could be due to different interpretations about the coefficients of the CCT models used in those studies^12^, a true understanding of the role of NDD in maintaining species diversity requires well-designed experiments on the mechanisms that regulate NDD. Currently, little is known about the mechanisms controlling relative abundances of species and especially how the mechanisms involved are related to the mechanisms maintaining species diversity.

One of the major mechanisms hypothesized to be widely responsible for maintaining species diversity in forests is specialized or at least partially specialized natural enemies, originally proposed independently by Janzen and Connell^10^,^12^,^13^,^14^,^15^,^16^,^17^,^18^,^19^,^20^,^21^,^22^. The key to this hypothesis is that host-specific pathogens or herbivores are maintained by or near adult trees, inhibiting the establishment and later success of conspecific seedlings in the vicinity. The inhibition of conspecifics thus releases space near adult trees favouring the establishment of heterospecifics. In principle, this effect leads to decreasing per capita recruitment as adult densities increase, in accordance with the idea of NDD discussed above.

Although Janzen and Connell both emphasized the importance of localization of natural enemies near adult hosts, in principle the mechanism would work with widely dispersing natural enemies that create no spatial structure in likelihood of a seedling being attacked, because NDD would still be present^23^. Moreover, some natural enemies may disappear altogether when adult densities are low, as is often observed in epidemiology^24^. Thus, a sufficiently low adult density might lead to very little effect of natural enemies even near a conspecific adult tree.

Although an increasing number of studies have supported the Janzen–Connell hypothesis as a primary density (or distance)-dependent mechanism regulating recruitment patterns of many tree species in forests as well as grassland ecosystems^10^,^12^,^13^,^14^,^15^,^16^,^17^,^18^,^19^,^20^,^21^,^22^,^25^,^26^,^27^,^28^, very few studies have actually isolated specific natural enemies and tested their effects on plant establishment^17^,^25^,^28^. For those studies that focused on soil pathogens and conducted fungicide treatments in the field, the experiments focused on species that were abundant at the experimental sites^19^,^27^, or no comparisons between population densities were made^25^. Here we performed a field survey and two growth-room experiments on *O. glaberrima* with soil collected from six field sites varying in tree density from rare to abundant. We asked whether the Janzen–Connell mechanism is operating on two scales: in the vicinity of an adult and between sites differing greatly in density. We thus tested whether the mechanism is sensitive to adult density on a larger scale, not merely in the vicinity of a parent tree of a given seedling. The results showed that the survival of *O*. *glaberrima* seedlings in low-density population sites was high compared with that in abundant population sites. Soil-borne fungal pathogens were found to be responsible for suppressing seedling recruitment of *O. glaberrima* when it is abundant but had little adversary effect on the seedlings when it is rare, owing to the lack of pathogens at the low host-density sites.

## Results

### Seedling survival observed from the field

**Figure 1:**
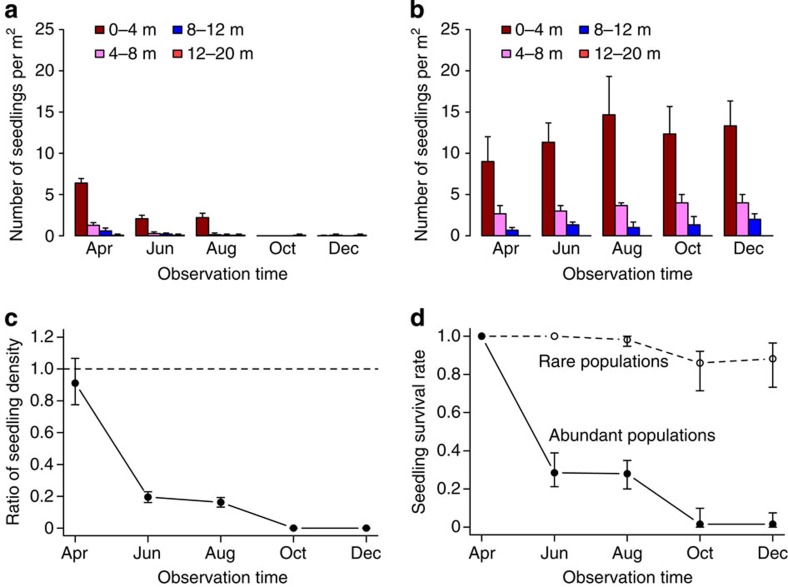
Effects of seedling density over time (month) and distance on seedling survival. (**a**) Changes in seedling density over time and distance for abundant population sites. (**b**) Changes in seedling density over time and distance for low-density population sites. (**c**) Ratios of seedling density (within 0–8 m from the focal trees, where over 90% seedlings located) of abundant sites over the density of low-density sites. The dashed line refers to equal seedling density between abundant and low-density sites. (**d**) Changes in per-capita seedling survival rates over time within 0–8 m from the focal trees. The number of seedlings at each observation time was the old seedlings that survived up to that time plus the new seedlings that germinated since the previous time. The per-capita seedling survival rate was calculated by dividing the number of live seedlings at an observation time by the total tagged seedlings up to that time. In **c**, the interval for the ratio of seedling density in April 2012 contains 1, suggesting no significant difference in density between abundant and low-density populations at the beginning of observation. All the error bars are 95% bootstrapped confidence intervals.

The field results showed that the densities of surviving *O. glaberrima* seedlings at different distances from adult trees in abundant and low-density population sites fared very differently (Fig. 1). In the abundant sites, seedling density decreased dramatically over time with few survival seedlings left after August (Fig. 1a). Moreover, the distance effect disappeared at distance larger than 8–12 m away from the adult trees. In contrast, seedling density changed little over time for the three low-density sites (Fig. 1b). The contrasting difference between abundant and low-density populations is clearly revealed by the decreasing ratios of their seedling densities (Fig. 1c) and by the trend of per-capita seedling survival rates over time (Fig. 1d). We did a non-parametric Mann–Whitney test to compare the difference in seedling density within 8 m of focal trees between the abundant and low-density populations for each of the five months (that is, testing the difference between Fig. 1a,b for the pooled density for 0–8 m). All the *P*-values of the tests are at least smaller than 0.0069 (sample sizes: *n*_1_=15 abundant sites versus *n*_2_=3 low-density sites), except for April when field observations started (*P*=0.37; sample size: *n*_1_=15 versus *n*_2_=3), indicating highly significant differences between the two populations.

We further modelled the effects of abundance, distance and their interaction on seedling survival rates using a generalized linear mixed effects model (GLMM; see ‘Statistical analysis' in Methods). The results do not only show significant effects of abundance and distance but also their interaction on seedling survival (Table 1). If survival rates were the same between the abundant and low-density sites, the abundance term would not be significant. The results in Table 1 show that abundance had a highly significant negative effect on seedling survival (abundant sites were coded as 0 and low-density sites as 1), while distance had a positive effect. The significant interaction term between abundance and distance indicates that distance had very different effects on seedling survival rates between the abundant and low-density sites, which is consistent with the results of the above Mann–Whitney test. Together, these results indicate that soil-borne pathogens suppressed the recruitment of *O. glaberrima* seedlings in the abundant sites but had no detectable effect on seedlings in the low-density sites.

### Effects of pathogens in growth-room experiments

**Figure 2:**
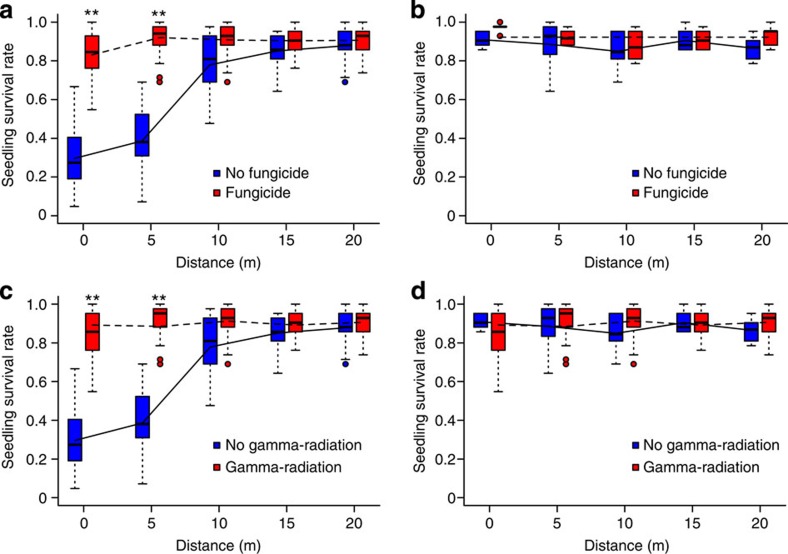
Distance effect from focal trees on seedling survival. Upper panels are the results for fungicide-treated soils, whereas lower panels are for γ-radiation-treated soils, to show the distance effect from focal trees on seedling survival. (**a**,**c**) Soil samples were collected from abundant sites; (**b**,**d**) soil samples were collected from low-density sites. Boxplots for untreated soil are in blue colour (solid line) and red colour (dashed line) for fungicide-treated soil (**a**,**b**) or γ-treated soil (**c**,**d**). Each boxplot shows the median, inter-quartiles and whiskers. Dots outsides the whiskers are outliers. **indicates seedling survival rates in fungicide (or γ-radiation)-treated soils were significantly higher than that in untreated soils collected at the respective distances of 0 and 5 m away from the focal trees in the abundant sites. The *P*-values for the two ** in **a** are 6.27e−11 and 2.87e−11, respectively (Mann–Whitney test), and for the two ** in **c** are 6.27e−11 and 2.86e−11, respectively. Sample size for each of the Mann–Whitney tests is 90 untreated soils versus 90 treated soils.

When seedling survival was tested in untreated soils collected at different distances from focal trees of the three abundant sites (see Methods for the growth-room experiment), seedling survival significantly increased with increasing distance from the focal trees (Fig. 2a). However, this trend was not observed in the soils collected from the three low-density sites (Fig. 2b). After fungicide treatment, the effect of distance on seedling survival disappeared. A similar result was also observed for γ-radiation-treated soils (lower panels in Fig. 2). This result is consistent with the result of the GLMM modelling for the growth-room experiment (see ‘Statistical analysis' in Methods), showing seedling survival increased with distance in the abundant populations (Table 2; the highly significant three-way interaction of abundance, distance and fungicide, *P*=4.22e−07) and local seedlings of a focal tree suffered a significantly higher mortality from soil-borne pathogens than non-local seedlings derived from seeds collected from other trees (*P*=4.46e−08 for ‘Seed' in Table 2; by odds ratio the likelihood of survival of non-local seedlings is *e*^0.46^=1.58 times higher than that of local seedlings, if else is equal). All these results suggest that disease-inducing fungi are the primary agent suppressing seedling recruitment in abundant *O. glabrrima* sites. It is noteworthy that there was no effect of planting density on seedling survival (*P*=0.45 in Table 2) and the variance of the random effect ‘Site' was not different from 0 (*P*=1 in Table 2).

**Figure 3:**
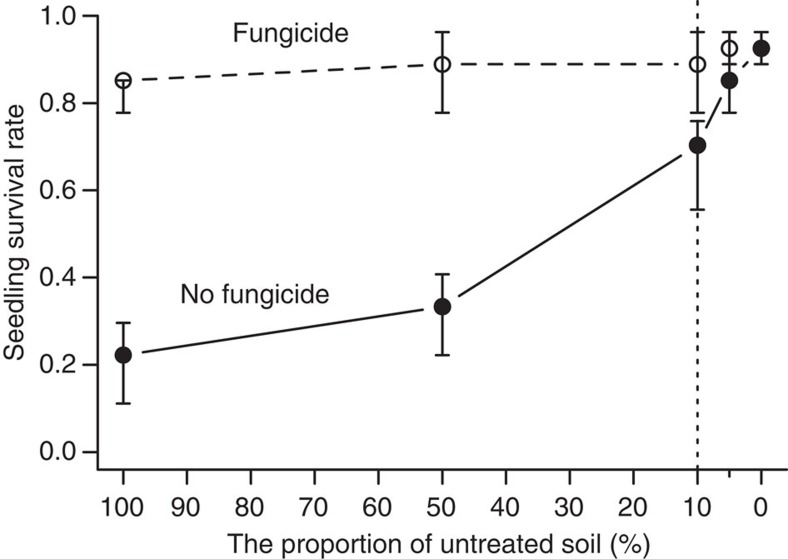
Effects of the proportion of untreated soil on seedling survival of *O. glaberrima*. The soil used for the experiment was a mixture of untreated soil and γ-radiated soil in different proportions. This dilution experiment was only performed with local *O. glaberrima* seeds. For comparison, two fungicides were further applied to the diluted soil, to remove the effect of inoculating untreated soil (the open dots and dashed line). When the proportion of untreated soil is <10% (to the right of the vertical dotted line), seedling survival rates are no longer different from that of fungicide-treated soils (for example, at 5% untreated soil, Mann–Whitney test: *P*=0.30; sample size: *n*_1_=3, *n*_2_=3). The error bars are 95% bootstrapped confidence intervals.

Seedling survival in a dilution experiment increased with the reduction of untreated soil from abundant sites (see Methods for dilution experiment), whereas there was little change in seedling survival (consistently high) after the soil was treated with two fungicides (Fig. 3). When the proportion of untreated soil was <10%, the survival of *O. glaberrima* seedlings was no longer affected by inoculation with untreated soil (for example, at 5% untreated soil, Mann–Whitney test had *P*=0.30 with sample size *n*_1_=3 versus *n*_2_=3; Fig. 3).

## Discussion

In this study we performed field observations and growth-room experiments to test the effects of soil-borne pathogens on abundant and low-density populations of a subtropical tree species. Our results show that soil pathogens significantly suppressed the recruitment of *O. glaberrima* in sites where the host populations were abundant but had little effect on the low-density populations. The seedling survival rates in low-density population sites were significantly higher compared with those in abundant sites (Fig. 1 and Tables 1 and 2). However, this increased survival in low-density populations was caused by the lack of pathogenic effects as shown by both the field observation (Fig. 1) and the growth-room sterilized treatments (Figs 2 and 3). The density of soil pathogens such as *Fusarium oxysporum*^28^ in all low-density sites was too low to cause disease to the seedlings of the study tree species (Fig. 3), where seedling survival was nearly as high as that in the fungicide-treated or γ-radiated soils (Fig. 2b,d). In contrast, seedling survival was significantly reduced within approximately a 10-m vicinity of the focal trees in the abundant sites but the survival rates became as high as the fungicide (or γ-radiation)-treated level at a distance >10 m from the focal trees (Fig. 2a,c). If measured by odds ratio, assuming all else is equal, the likelihood of seedling mortality in abundant populations is *e*^9.64^=15367.3 times higher than that in low-density populations in the field (Table 1) and *e*^3.27^ =26.3 times higher in the growth-room experiment (Table 2). However, it is worth noting that adult trees in abundant sites do not necessarily show uniform pathogenic effects possibly arising from the spatial variation in pathogen distribution or in the local microhabitat conditions. This pathogenic variation is reflected by the wide variation in seedling survival rates at 0 and 5 m distances for trees in abundant sites (Fig. 2a,c).

This study has demonstrated the Janzen–Connell mechanism on two scales: within sites based on distance from an adult tree and between sites based on site level contrasts in density. In both cases, the proximate mechanism for seedling mortality appears to be pathogen abundance, which on the between-site scale is strongly affected by conspecific adult abundance, but on the within-site scale the distance effect was only observed in abundant sites. As such, there is an interaction between the two scales: reduction in pathogenicity at low-density sites is associated with the absence of a within-site effect of distance from a conspecific tree. This outcome suggests that a host density above some threshold is necessary to maintain the relevant pathogens on the site scale similar to the behaviour of disease in epidemiological studies^24^.

**Figure 4:**
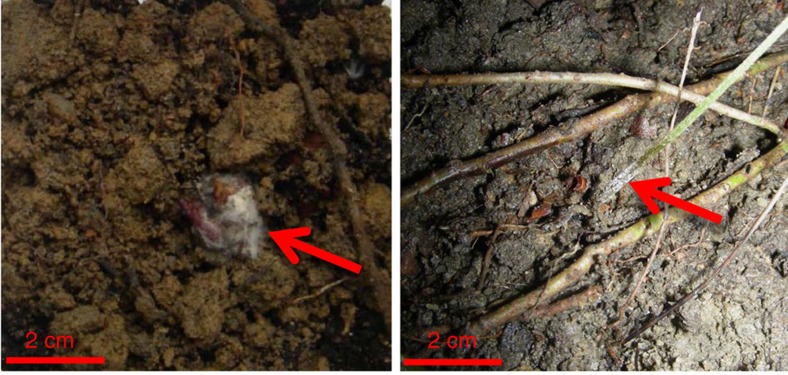
Symptoms of a rotten seed and an infected seedling of *O. glaberrima*. Left photo shows an infected seed with filaments of pathogenic fungi including *F. oxysporum* in the soil collected within 5 m from focal adult trees in abundant sites, which were found to contain the pathogen infesting *O. glaberrima*. Right photo shows an infected seedling (damping-off symptom) with lower part of stem covered with fungal spores in the abundant sites. Image credit: Yu Liu.

Our experiments also imply that the relevant pathogens are host specific, which is a key feature of the Janzen–Connell mechanism. The identification of *F. oxysporum* as a pathogen for *O. glaberrima*^28^ supports this conclusion. In an inoculation experiment, this fungus caused rapid death of *O. glaberrima* seedlings (see rotten seed and sick seedling in Fig. 4) but failed to infect three other common tree species of different families that grow with *O. glaberrima* in the same sites, namely *Castanopsis fabri*, *Cryptocarya concinna* and *Schefflera octophylla*^28^. Although we cannot rule out the possibility that the pathogen might also infest other tree species, the immunity of the three common tree species to *F. oxysporum* suggests a high degree of host specificity of the pathogen to *O. glaberrima*. The dilution experiment shown in Fig. 3 indicates that the survival of *O. glaberrima* seedlings was no longer affected by the pathogen inoculum if the proportion of untreated soil was approximately lower than 10%.

Although this study has exploited naturally occurring major density differences between sites to demonstrate the density dependence of pathogen effects, less clear is the origin of this between-site variation in density. We found that seedlings are not limited by pathogens in low-density sites, but that outcome does not appear to be translating into an overall low-density advantage, and increasing low populations. No juveniles and only one premature tree were found in the three low-density sites (Table 3) despite current high initial seedling densities and seedling survival rates in the sites (Fig. 1b). This finding suggests that most seedlings of *O. glaberrima* in low-density sites fail to reach the juvenile or premature tree stages. Seedling recruitment is the first of the many bottleneck processes a species population has to overcome to establish in a community^12^. The lack of the fungal pathogen effect in low-density population provides an initial fitness advantage for the species when at low density. However, a high seedling survival is necessary but not sufficient to ensure the subsequent establishment of the population. It seems likely to be that some environmental factors in these low-density sites are limiting success beyond the seedling stage but at the present time we can only speculate on what it might be. Whatever the mechanisms, the outcome has been the maintenance of low-density sites enabling a between-site adult density contrast in what is in effect a natural experiment.

Study of the Janzen–Connell hypothesis has so far been a mixture of both theories and empirical tests. A large number of studies have modelled and analysed spatial patterns or temporal dynamics of demographic rates of tree species^9^,^11^,^29^,^30^. These studies do not actually test for the Janzen–Connell hypothesis but use the hypothesis to interpret the patterns or dynamics observed. However, in the literature there is a widespread misunderstanding on these analyses and they are cited as if they tested the Janzen–Connell hypothesis. Before now, experimental evidence has not been available to evaluate the strength of the Janzen–Connell effect in tree populations beyond the seedling stage.

Another controversy arises regarding the observations that rare species suffer stronger negative density-dependent effects than common species do^9^,^10^,^11^. This is probably due to the use of different modelling methods or the interpretation of the models^12^. These studies compare the variation in demographic rates among species with varying abundance and their response to specialized enemies^10^, but the significance of such an interspecific comparison could be compromised by the fact that different species can have different co-evolutionary history with their own host-specific pathogens and, as such, more common species do not necessarily suffer from stronger or weaker pathogen attack than rarer species. For example, Dostál *et al*.^31^ found that the effect of soil pathogens on an invasive species *Heracleum mantegazzianum* (Apiaceae) depended on the invasion history of the species^31^.

Our study did not consider interspecific variation in demography but intraspecific variation. Although we did not directly test the CCT hypothesis, we showed how NDD can emerge for a species, which is a prerequisite of the CCT^2^,^7^,^8^. If there were no NDD on the individual species level, there would be no CCT on the community level. Our results support the NDD on two spatial scales, giving as strong an affirmation of NDD as might be expected from natural contrasts in densities. Our results support the idea that the observed NDD arises from a pathogen that is weakened by low host density. Although we have not identified the factors responsible for our low-density sites, they are not necessarily explained by a density-dependent factor and so are not support for stronger NDD for rare species. We argue that manipulative experiments are urgently needed to extend the current study to test interspecific responses to specialized pathogens, to identify and understand the mechanisms of the CCT in maintaining species diversity in communities. Our results suggest also that approaches combining the effects of physical environmental factors with studies of pathogens are needed to understand major spatial differences in abundance and a better perspective on species' rarity.

## Methods

### Study sites

The sites are located in the Heishiding Nature Reserve, a subtropical, evergreen broadleaf forest, China (N23°27′12″, E111°53′26″). *O. glaberrima* Wu (Fabaceae) is a native legume tree common to the region. Its seeds are dispersed by gravity in late September and >90% seeds (based on our visual estimation in the field) fall within 8 m from their parent trees. After one-year storage, *O. glaberrima* seeds still have a high germination rate (94.22±2.61%). Three low-density and three abundant population sites (1-ha plot each) of this species were selected in March 2012, including the two sites studied in ref. 28. A low-density site had a density of ∼1 adult tree per hectare, whereas an abundant site had a tree density >20 adult trees per hectare (Table 3). The distances between these six populations were at least 1 km apart. In each site, all individuals of this species with diameter at breast height ≥1 cm were mapped. In the meantime, seeds were collected from the ground from these six populations for the growth-room experiments (described below).

In each of the three abundant sites, five adult *O. glaberrima* trees with similar diameter at breast height sizes were selected as focal trees, while a single adult *O. glaberrima* tree was selected as a focal tree in each of the three rare sites. A 1 × 20 m seedling belt was established away from each focal tree in such a way that the focal tree was the nearest conspecific adult to any point within the seedling belt. The belt was divided to five segments: 0–4, 4–8, 8–12, 12–16 and 16–20 m (but because there were very few seedlings at distance >12 m, the data of the last two segments were combined in subsequent analyses). Most seeds of *O. glaberrima* started to germinate in early April and completed germination within the first three weeks of April, although they could germinate in other months. Newly emerged seedlings were counted and tagged with unique numbers in each segment. The survival status of all tagged seedlings was recorded at the end of each month from April to December 2012.

### Growth-room experiment

Next to each seedling belt, soil samples from the top soil (*ca.* 10 cm in depth) were collected at 0, 5, 10, 15 and 20 m away from a focal tree in March 2012 (corresponding to the five segments of each belt). Soil samples were sieved (mesh diameter: 0.2 cm) to eliminate seeds. In total, 90 soil samples (for abundant populations: 3 populations × 5 focal trees per population × 5 distances=75; for low-density populations: 3 populations × 1 focal tree per population × 5 distances=15) were shipped to a growth room at the Guangzhou Institute of Landscape Gardening for growth-room experiments. Each soil sample was divided into three parts: untreated, fungicide-treated (a mixture of two fungicides Celest Gold and Ridomil Gold; Syngenta Ltd, Basel, Switzerland) and γ-radiated. Celest Gold (active ingredient: fludioxonil) offers the best control for *Fusarium* spp. and *Microdochium nivale*. Ridomil Gold (active ingredient: mefenoxam) preferentially acts against fungus-like oomycetes and has been applied in previous studies^18^,^32^.

Seed provenance of *O. glaberrima* was divided into two types (local seed: seeds planted in soil sampled from the same population where seeds were collected; non-local seed: seeds mixed from the other five populations). The local and non-local seeds were cross-planted in soils from focal trees and non-focal trees, to test the consequences of co-adapted soil pathogens for seed flow^33^. In total, there were 180 cross-plantings (90 × 2=180).

After treatment with 98% concentrated sulfuric acid for 30 min (for sterilizing seed surface and breaking physical dormancy of seeds), seeds were transferred into a 250-ml soil sample in a sterilized 12 × 12 × 20 cm plastic vessel (upper vessel), which was linked with a cotton wick to a similar vessel (lower vessel containing sterilized water). A 1-cm layer of sterilized expanded clay (*ca.* 1 mm in diameter) was covered onto the soil sample to block ambient microbes^28^.

The planting experiment set three densities (low density: one seed per vessel, intermediate density: four seeds per vessel and high density: nine seeds per vessel) and three replicates per density treatment. All the experiments were carried out in a growth-room with 24±2 °C, 80–95% relative humidity and low-light ambient conditions (*ca.* 8% of outdoor light intensity in the daytime), similar to conditions under canopy. The growth-room experiment lasted from April to December 2012 and seedling survival was recorded every 2 weeks.

### Dilution treatment

We tested whether a decreasing pathogen concentration in the soil caused the increased seedling survival observed in the growth-room experiment. In this dilution experiment, done in the same facility as for the above growth-room experiment, untreated soil sampled around the focal adult *O. glaberrima* trees (<5 m) from abundant sites was mixed, at different ratios, with soil sampled from the same location but γ-radiated. In total, five dilution ratios were tested and the ratios of untreated soil to γ-radiated soil by volume were 100:0, 50:50, 10:90, 5:95 and 0:100. The mixed soil was then divided into two halves. One was further treated with the two aforementioned fungicides and the other half was without. Three replicated experiments were conducted for each half. The effects of the dilution treatment on seed germination and seedling survival were observed biweekly from April to December 2012.

### Statistical analysis

To analyse the data of the field survey (see Table 1 for the results), a GLMM was used to test for the effects of adult abundance (abundant versus low-density populations), distance from focal trees and the interaction term (abundance × distance) on seedling survival of *O. glaberrima*. Population sites were treated as a random effect, to control for the variation in seedling survival among sites. The dependent variable of the GLMM was the count of dead and live seedlings by the end of the field observation (that is, December 2012). The live seedlings were those that survived up to December 2012 from the beginning of field observation in April, whereas the dead seedlings were those dead during the period. The per-capita seedling survival rate in a month as shown in Fig. 1d was calculated by dividing the number of live seedlings survived up to that month by the total of the emerging seedlings (live+dead) from April up to that month. A similar GLMM (see Table 2 for the results) was also used to model seedling survival data obtained from the growth-room experiment, to test for the effects of adult abundance (abundant versus low-density populations), distance from focal trees, soil fungicides treatment, seed provenance (local versus non-local seeds) and seeding density on seedling survival. Population sites were treated as a random effect. Both of the above GLMM models assumed a binomial error and overdispersion was included in these two GLMMs by estimating an additional random effect for each observation^34^,^35^.

All the data in this study were analysed using R (http://www.r-project.org/). The GLMMs were estimated using R package ‘lme4'. The 95% confidence intervals reported in this study are the bootstrap-accelerated bias-corrected confidence intervals calculated from R package ‘boot'.

## Additional information

**How to cite this article:** Liu, Y. *et al*. The effect of soil-borne pathogens depends on the abundance of host tree species. *Nat. Commun.* 6:10017 doi: 10.1038/ncomms10017 (2015).

## Figures and Tables

**Table 1:** GLMM for modelling seedling survival data from the field.

Fixed effects	Estimated coefficients (s.e.)	*z*-value	*P* (>|*z*|)
(Intercept)	−7.46 (1.05)	−7.13	1.03e−12
Abundance	9.64 (1.30)	7.04	1.35e−13
Distance	0.46 (0.093)	4.89	9.61e−07
Abundance × distance	−0.52 (0.13)	−3.90	9.52e−05
**Random effect**	**Variance**		***P*** **(>*****χ***^**2**^)
Site (intercept)	0.14		0.53

GLMM, generalized linear mixed effects model.

A GLMM was used to test the effects of adult abundance (0 for abundant populations and 1 for low-density populations), distance (0–4, 4–8, 8–12 and 12–20 m from a focal tree) and their interaction on seedling survival of *O. glaberrima*. Population sites were treated as a random effect. The dependent variable of the GLMM was the count of dead and live seedlings by the end of the field observation (that is, December 2012). The number of seedlings at this time (December 2012) was the live seedlings that survived up to this time plus the new seedlings that germinated since the previous time (that is, October 2012). The estimated overdispersion variance was 0.55.

**Table 2:** GLMM for modelling seedling survival data from the growth-room experiment experiments.

Fixed effects	Estimated coefficients (s.e.)	*z*-value	*P* (>|z|)
(Intercept)	−1.60 (0.17)	−9.44	<2e−16
Abundance	3.27 (0.22)	14.88	<2e−16
Density	0.011 (0.014)	−0.76	0.45
Distance	0.18 (0.0068)	25.96	**<**2e−16
Fungicide	3.58 (0.26)	13.53	**<**2e−16
Seed	0.46 (0.084)	5.47	4.46e−08
Abundance × distance	−0.19 (0.018)	−10.76	**<**2e−16
Abundance × fungicide	−2.38 (0.34)	−7.00	2.59e−12
Distance × fungicide	−0.15 (0.010)	−14.81	**<**2e−16
Fungicide × density	−0.0087 (0.022)	−0.40	0.69
Fungicide × seed	−0.34 (0.13)	−2.63	0.0086
Abundance × distance × fungicide	0.14 (0.027)	5.06	4.22e−07
**Random effect**	**Variance**		***P*** **(>*****χ***^**2**^)
Site (intercept)	1.68e−06		1.00

GLMM, generalized linear mixed effects model.

A GLMM was used to test the effects of adult abundance (0 for abundant populations and 1 for low-density populations), distance (0, 5, 10, 15 and 20 m from a focal tree), seeding density (1, 4 and 9 seeds per vessel), seed (0 for local seeds and 1 for nonlocal seeds), soil treatment (0 for no fungicide and 1 for fungicide) and interaction terms (for example, abundance × fungicide treatment) on seedling survival of *O. glaberrima*. The dependent variable of the GLMM was the count of dead and live seedlings by the end of the growth-room experiment. The estimated overdispersion variance was 0.33.

**Table 3:** Population structures (by DBH classes) of *O. glaberrima* trees in the three abundant populations (A1–A3) and the three rare populations (R1–R3).

Abundance	Population	Juvenile (DBH: 1–9 cm)	Premature (10–25 cm)	Mature (>25 cm)
Abundant	A1	32	14	26
	A2	20	16	30
	A3	36	15	22
Low density	R1	0	0	1
	R2	0	0	1
	R3	0	1	1

DBH, diameter at breast height. The division of DBH classes followed ref. 36. No juvenile and only one premature tree was found in the three low-density population sites, suggesting chance for seedlings in the low-density sites to reach the juvenile, premature or mature stage was very low, although more newly germinated seedlings survived in the low-density sites than in the abundant sites and per-capita survivorship at the low-density sites was also higher than at the abundant sites.
